# TGFβ superfamily signaling and uterine decidualization

**DOI:** 10.1186/s12958-017-0303-0

**Published:** 2017-10-13

**Authors:** Nan Ni, Qinglei Li

**Affiliations:** Department of Veterinary Integrative Biosciences, College of Veterinary Medicine and Biomedical Sciences, Texas A&M University, College Station, TX 77843 USA

**Keywords:** TGF-beta, Activin, BMP, SMAD, TGFBR1, Decidualization

## Abstract

Decidualization is an intricate biological process where extensive morphological, functional, and genetic changes take place in endometrial stromal cells to support the development of an implanting blastocyst. Deficiencies in decidualization are associated with pregnancy complications and reproductive diseases. Decidualization is coordinately regulated by steroid hormones, growth factors, and molecular and epigenetic mechanisms. Transforming growth factor β (TGFβ) superfamily signaling regulates multifaceted reproductive processes. However, the role of TGFβ signaling in uterine decidualization is poorly understood. Recent studies using the Cre-LoxP strategy have shed new light on the critical role of TGFβ signaling machinery in uterine decidualization. Herein, we focus on reviewing exciting findings from studies using both mouse genetics and in vitro cultured human endometrial stromal cells. We also delve into emerging mechanisms that underlie decidualization, such as non-coding RNAs and epigenetic modifications. We envision that future studies aimed at defining the interrelationship among TGFβ signaling circuitries and their potential interactions with epigenetic modifications/non-coding RNAs during uterine decidualization will open new avenues to treat pregnancy complications associated with decidualization deficiencies.

## Background

**Fig. 1:**
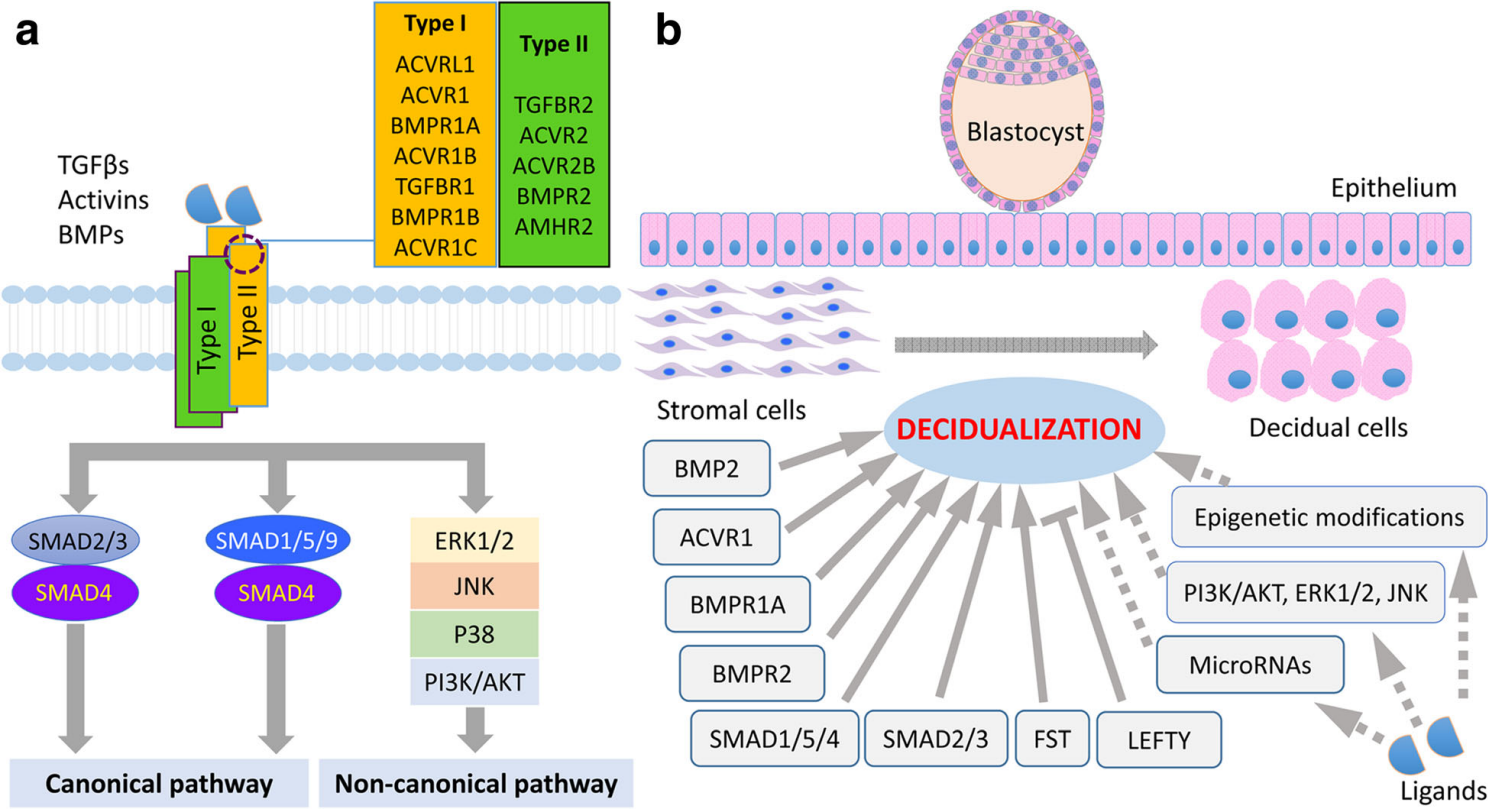
Schematic illustration of TGFβ superfamily signaling and its involvement in decidualization. **a** A diagram of TGFβ superfamily signaling. TGFβ superfamily ligands (e.g., TGFβs, activins, and BMPs) induce the formation of membrane-associated receptor complexes comprising type 1 and type 2 receptors. Activated receptor machinery phosphorylates SMAD proteins (i.e., SMAD2/3 and SMAD1/5/9), which cooperate with SMAD4 to function in a canonical pathway. The non-canonical pathways generally include, but are not limited to, ERK1/2, JNK, P38, and PI3K/AKT, the activation of which is SMAD-independent. **b** TGFβ signaling components and uterine decidualization. Experimental evidence, particularly those from genetically modified mouse models, has revealed critical functions of various TGFβ signaling elements in the process of uterine decidualization. Disruption of BMP2, ACVR1, BMPR1A, BMPR2, SMAD1/5/4, SMAD3, or FST leads to defects in uterine decidualization. In contrast, LEFTY seems to be a suppressor of uterine decidualization. Further clarification of the function of TGFβ superfamily ligands (e.g., TGFβs and activins) and the usage of type 1 and type 2 receptors by different signaling molecules is warranted. Studies are also needed to assess the role of the non-canonical TGFβ signaling branch in decidualization and potential interactions between TGFβ superfamily signaling and epigenetic modifications and microRNAs in this key remodeling event. As decidualization is a highly orchestrated process regulated by hormonal, cellular, and molecular mechanisms, this diagram focuses on highlighting molecules associated with the TGFβ signaling pathway

Transforming growth factor β (TGFβ) superfamily proteins regulate a variety of cellular functions via serine/threonine kinase receptors and SMAD proteins [1]. More than 40 members of TGFβ superfamily ligands have been identified, which include TGFβs, bone morphogenetic proteins (BMPs), anti-Müllerian hormone (AMH), activins and inhibins, growth differentiation factors (GDFs), and nodal growth differentiation factor (NODAL) [2]. The ligand-receptor interaction induces a signal transduction cascade, where the type II receptors (i.e., TGFBR2, ACVR2, ACVR2B, BMPR2, and AMHR2) activate functionally related type I receptors (i.e., ACVRL1/ALK1, ACVR1/ALK2, BMPR1A/ALK3, ACVR1B/ALK4, TGFBR1/ALK5, BMPR1B/ALK6, and ACVR1C/ALK7) via phosphorylation. The activated TGFβ receptor complexes interact with intracellular receptor-regulated SMADs (R-SMADs), which are then associated with SMAD4 to gain access to nuclear transcriptional machinery and modulate gene transcription. In addition to the well-described canonical SMAD-dependent signaling branch, TGFβ superfamily members also utilize diverse pathways independent of SMAD transcription factors [3] (Fig. 1a).

Growing evidence has demonstrated the involvement of TGFβ signaling in many fundamental reproductive events highlighted below. (i) Folliculogenesis. TGFβ superfamily signaling regulates follicle growth and activation [4]. Some oocyte-derived TGFβ superfamily growth factors are obligatory for follicular development [1]. It also appears that these growth factors are important regulators of oocyte quality, evidenced by enhanced developmental potential of in vitro matured oocytes supplemented with recombinant oocyte-produced TGFβ family proteins such as GDF9 and BMP15 [5, 6]. (ii) Ovulation. Ovulatory defects have been observed in mice lacking SMAD4, SMAD2/3, or activin/inhibin subunits [7–11]. Several elegant reviews are available on the topic of TGFβ signaling in follicular development and ovulation [12–16]. (iii) Maternal-embryo communication. Maternal-embryo interactions are of critical importance for a successful pregnancy. TGFβ proteins have been suggested to play a role in the maternal-fetal interface during pregnancy [17, 18]. A recent study revealed a role for BMP signaling in mediating crosstalk between bovine embryos and the oviduct during early developmental stages, where the embryo-oviduct interactions alter BMP signaling differentially within oviductal cells and embryos [19]. (iv) Embryonic development. TGFβ superfamily members are implicated in the development of preimplantation embryos. A role for BMP4 and inhibitor of DNA binding 3 (ID3) has been suggested in the regulation of embryo development in the bovine [20]. TGFβ1 mRNA is expressed in fertilized mouse oocytes and blastocysts [21]. Moreover, BMP signaling activity is detectable in mouse embryos as early as the 4-cell stage and is needed for the cleavage of preimplantation embryos [22]. Besides its role in preimplantation embryonic development, TGFβ superfamily signaling is required for multiple developmental events in post-implantation embryos, such as patterning and gastrulation [23–25]. (v) Reproductive tract morphogenesis and function. TGFβ superfamily signaling regulates reproductive tract formation [26–28]. We have revealed that conditional knockout (cKO) of *Tgfbr1* in the female reproductive tract using anti-Müllerian hormone receptor type 2 (*Amhr2*)-Cre leads to the development of oviductal diverticula, myometrial defects, and infertility [29, 30]. We have also identified a potential role for TGFBR1-mediated signaling in regulating uterine epithelial cell function [31]. (vi) Decidualization. The role of TGFβ superfamily signaling in uterine decidualization is discussed in the following section. Table 1 lists major functions of the TGFβ superfamily in reproduction and development along with some important signaling components that are involved in the regulation of those functions.

**Table 1:** Major roles of TGFβ superfamily in reproduction and development

Reproductive event	Main signaling component	Reference
Folliculogenesis	TGFβs, GDF9, BMP2, BMP4, BMP7	
Oocyte maturation	BMP15, activin, inhibin, AMH	[1, 4–16, 61, 105]
Ovulation	BMPR1A, BMPR1B, SMAD2/3, SMAD4	
Maternal-embryo interactions	TGFβs, BMP7, BMPR1B, BMPR2, SMAD1, SMAD6	[17–19]
Implantation	TGFβ1, ACVR1, BMPR1A, TGFBR1, BMP7	[52, 59, 64, 106]
Decidualization	BMP2, SMAD1/5/4, SMAD2/3, ACVR1, BMPR1A, BMPR2, FST, LEFTY	[41, 54, 57–60, 63, 67, 69]
Embryonic development	TGFβs, BMP2, BMP4, BMP5, BMP6, BMP7, BMP8, INHBA, INHBB, GDF1, LEFTY, NODAL, AMH, SMAD1, SMAD2, SMAD4, SMAD5, SMAD6, SMAD7, ACVRL1, ACVR1, BMPR1A, ACVR1B, TGFBR1, BMPR1B, TGFBR2, ACVR2/2B, AMHR2, BMPR2	[20–25, 107, 108]
Reproductive tract development	TGFBR1, AMH, AMHR2, ACVR1, BMPR1A, SMAD1/5/8, SMAD4	[29, 30, 109, 110]

It is important to note that, in addition to its role in female reproductive function, TGFβ signaling also regulates the development and function of the male reproductive system such as testis development [32]. However, this topic is beyond the scope of this review.

## Uterine decidualization: A critical event during pregnancy

A successful pregnancy relies on a delicate interplay among hormonal, cellular, and molecular signals. Decidualization, a process where extensive remodeling of the endometrium occurs to set the stage for embryo development, is a key event in pregnancy in some mammals including mice and humans. Despite its critical role in pregnancy, the timing of decidualization differs among species. Decidualization is induced by attachment of the blastocyst to uterine luminal epithelium in mice, whereas differentiation of the estradiol (E2)-primed endometrium occurs following the postovulatory rise of progesterone (P4) during the secretory phase of menstrual cycle in humans [33, 34]. During decidualization, dramatic cellular and molecular changes occur, as endometrial stroma cells (ESCs) transform from fibroblast-like cells into large polygonal cells that are rich in cytoplasmic glycogen and lipid droplets [35]. Stromal cell polyploidy is a unique phenomenon that occurs during decidual cell differentiation following blastocyst implantation [34]. Decidual cell-secreted factors include prolactin (PRL) and insulin-like growth factor binding protein-1 (IGFBP-1) that are key regulators of decidualization and are widely used as markers of decidualization [36, 37].

Ovarian steroid hormones, E2 and P4, play fundamental roles in implantation of blastocysts and uterine decidualization [38]. It has been increasingly recognized that progesterone receptor (PGR) signaling is of paramount importance for blastocyst implantation, uterine decidualization, and pregnancy maintenance [38, 39]. P4, via binding to its cognate receptor, activates a complex array of molecular events mediated by Indian hedgehog (IHH) [40], BMP2 [41], nuclear receptor subfamily 2 group F member 2 (NR2F2/COUP-TFII) [42], Wingless-type MMTV integration site family (WNT) 4 [43], and HAND2 [44] during implantation and/or decidualization. Examples of additional PGR-associated regulators of endometrial function include forkhead box O1 (FOXO1) [45], CCAAT/enhancer-binding protein beta (CEBPB) [46], and homeobox A10 (HOXA10) [47, 48]. Of note, immune cells, particularly uterine natural killer (uNK) cells, can be recruited to regulate important events such as decidual angiogenesis during pregnancy [49].

In the following sections, we review literature that documents a role for TGFβ superfamily signaling in uterine decidualization, with a focus on major findings from genetically modified mouse models and cell culture studies using human ESCs.

## TGFB superfamily signaling regulates uterine decidualization

### Evidence from mouse models

#### TGFβ superfamily ligands

The role of TGFβ ligands in uterine decidualization in mice is not clear. Existing evidence suggests that TGFβ signaling pathway may be involved in regression of the uterine decidua in the rat, as TGFβ1, TGFβ2, TGFβ3 are highly expressed during the regression of the decidua basalis, accompanied by an upregulation of expression of phosphorylated SMAD2 [50]. In vitro studies using rat decidual cells in culture revealed a role of TGFβ1 in inducing cellular apoptosis potentially through activation of SMAD2 and downregulation of AKT and X-linked inhibitor of apoptosis (XIAP) [50]. TGFβ2 and TGFβ3 also promote apoptosis in cultured decidual stromal cells potentially through regulation of AKT and XIAP expression [51].

Compelling evidence supporting an essential role for BMPs in uterine decidualization derives from studies using conditional deletion of *Bmp2* in the uteri of mice [41]. Loss of BMP2 in the uterus renders the mouse infertile and the uterus is unable to decidualize, owing to the dysregulation of multiple genes including *Wnt4/6*, FK-506 binding proteins (*Fkbps*), and prostaglandin synthase2 (*Ptgs2*) [41]. Recently, conditional deletion of *Bmp7* induces implantation defects and dysregulation of decidual genes including *Bmp2*, *Ptgs2*, *Wnt4*, and epiregulin (*Ereg*) [52]. However, *Bmp7* cKO mice respond normally to artificial decidualization stimuli [52]. Results of in vitro culture studies using undifferentiated uterine stromal cells from pregnant mice reinforce the role of BMP2-WNT4 signaling in decidualization [53]. In contrast to BMPs, the role of activins in uterine decidualization in mice remains elusive. However, a recent study has shown that follistatin (FST), an antagonist of activin, is required for blastocyst implantation and normal uterine decidualization [54].

NODAL, a key regulator of embryogenesis, is implicated in several pregnancy-associated events, including implantation of blastocyst and uterine decidualization [55]. Deletion of *Nodal* in the mouse uterus using *Pgr*-Cre leads to fertility defects, accompanied by fetal loss and preterm birth due to intrauterine growth restriction and malformation of the decidua basalis [56]. Of note, the NODAL antagonist, LEFTY, appears to inhibit uterine decidualization. Artificial decidualization of mice promotes the expression of LEFTY [57]. However, overexpression of LEFTY in the uteri of pregnant mice compromises artificial decidualization [58].

#### Receptors

The function of TGFβ superfamily signaling receptors in uterine decidualization is poorly understood, due in part to the promiscuity and redundancy of the receptor signaling. The application of Cre-LoxP approach to circumvent embryonic lethality of the receptor null mice greatly facilitates the dissection of the functional roles of TGFβ receptor signaling in the uterus.

BMPs generally signal through activin A receptor type 1 (ACVR1, also known as ALK2), BMP receptor type 1A (BMPR1A, known as ALK3), BMPR1B (known as ALK6), and BMP type 2 receptor (BMPR2). Conditional ablation of ACVR1 in the mouse uterus causes infertility, with delayed embryo invasion into the endometrium and defective implantation [59]. Expression of uterine stromal cell differentiation markers including *Prl8a2* and *Prl3c1* and the activity of alkaline phosphatase (ALP) are reduced in *Acvr1* cKO mice. Gene profiling using artificially decidualized uterine tissues identified CEBPB as a critical BMP downstream target [59]. Conditional deletion of *Bmpr1a* in the uteri of mice using *Pgr*-Cre leads to sterility [60]. *Bmpr1a Pgr*-Cre cKO mice manifest defective implantation and decidualization, with reduced expression of implantation-associated genes such as *Cox2* and *Wnt4*. Enhanced E2 signaling is evident in *Bmpr1a Pgr*-Cre cKO mice, where the expression levels of ER and its downstream signaling targets are higher than for controls [60]. Thus, BMPR1A-mediated signaling is critical for implantation and decidualization in mice. In contrast, conditional deletion of *Bmpr1a* using *Amhr2*-Cre leads to subfertility and a prolonged diestrous phase, without compromising decidualization [61]. Since *Amhr2*-Cre does not delete genes in uterine epithelia compared with *Pgr*-Cre, this finding suggests a potential involvement of epithelial BMPR1A in uterine decidualization. Knocking out of *Bmpr1b* results in infertility, accompanied by impaired cumulus expansion and uterine gland formation [62]. The uterine function of the type 2 receptor for BMPs, BMPR2, has been investigated via the creation of *Bmpr2* cKO mice using *Pgr*-Cre [63]. *Bmpr2* cKO mice are sterile, with intrauterine growth retardation and hemorrhaging observed in developing conceptuses (embryo/fetus and placenta) [63]. Unlike *Bmpr1a Pgr*-Cre cKO mice, the uteri of *Bmpr2* cKO mice are able to decidualize, although to a lesser extent than controls [63]. Interestingly, the number of uNK cells is substantially reduced in the decidua basalis of pregnant *Bmpr2* cKO mice [63]. Findings from *Bmpr2* cKO mice indicate that BMPR2-meidated signaling is not fully responsible for uterine decidualization.

In contrast to the reproductive phenotypes manifested by the aforementioned BMP signaling related mouse models, conditional ablation of TGFβ receptor 1 (TGFBR1, known as ALK5) leads to prominent defects in the female reproductive tract [29]. While the formation of myometrial layers is disrupted in *Tgfbr1 Amhr2*-Cre cKO mice, uterine decidualization can be induced artificially [29]. In contrast, *Tgfbr1 Pgr*-Cre cKO mice display defects in multiple pregnancy-related events including implantation, development of trophoblast cells, recruitment of uNKs, and uterine vascularization [64]. Of note, artificial decidualization occurs despite impaired recruitment of uNKs to the decidua and dysregulated expression of NK cell associated genes such as interleukin 15 (*Il-15*) [64].

Both activin A receptor type 1B (ACVR1B, known as ALK4) and ACVR1C (known as ALK7) mediate NODAL signaling that is essential for pregnancy [55, 65]. Ablation of ACVR1B in the uterus using *Pgr*-Cre results in defects in female fertility and placental development [66]. However, implantation and decidualization do not seem to be affected in these mice [66]. The role of ACVR1C in uterine decidualization has not been reported. The primary mouse models created to study the role of TGFβ signaling in uterine function are summarized in Table 2.

**Table 2:** Mouse models to study TGFβ superfamily signaling in uterine function

Mouse model	Phenotype	Reference
*Tgfbr1 Amhr2*-Cre cKO	Disrupted myometrial formation with occurrence of artificial decidualization	[29]
*Tgfbr1 Pgr-*Cre cKO	Defective placentation, impaired recruitment of uNK cells, with occurrence of artificial decidualization	[64]
*Bmp2 Pgr-*Cre cKO	Infertility with loss of decidualization	[41]
*Bmp7 Pgr-*Cre cKO	Defective implantation with normal response to artificial decidualization stimuli	[52]
*Fst Pgr-Cre* cKO	Defective uterine receptivity and decidualization	[54]
*Nodal Pgr-*Cre cKO	Malformation of decidua basalis with fetal loss and preterm birth	[56]
*Acvr1 Pgr-*Cre cKO	Infertility with defective implantation and decidualization	[59]
*Acvr1b Pgr-*Cre cKO	Defective placental development but normal occurrence of implantation and decidualization	[66]
*Bmpr1a Pgr-*Cre cKO	Impaired implantation and decidualization	[60]
*Bmpr1a Amhr2*-Cre cKO	Subfertility with prolonged diestrous phase and occurrence of decidualization	[61]
*Bmpr1b* KO	Infertility, impaired expansion of cumulus cells of oocytes and uterine gland formation	[62]
*Bmpr2 Pgr-*Cre cKO	Infertility, defective decidual vascularization and decidualization	[63]
*Smad3* KO	Impaired artificial decidualization	[67]
*Smad1/5/4 Amhr2*-Cre cKO	Defective oviductal and myometrial development, impaired implantation and decidualization	[69]

#### SMADs

SMADs are intracellular mediators of canonical TGFβ signaling. Recent studies begin to facilitate understanding of the role of SMAD proteins in the uterus. Artificial decidualization is moderately impaired in *Smad3* null mice [67]. A potential overlapping function between SMAD2 and SMAD3 in decidualization has been revealed; in vitro knockdown of *Smad2* using an siRNA approach reduces expression of prolactin-related protein in *Smad3* ^−/−^ decidual cells [67]. As a central mediator of the canonical TGFβ signaling pathway, SMAD4 transduces signals of both TGFβ/activin and BMP family members. However, the role of SMAD4 in the uterus remains elusive. Uterine specific ablation of SMAD4 is expected to provide insight into its role. The role of BMP-associated SMADs in the uterus has been investigated. Conditional deletion of *Smad1* and *Smad5* using *Amhr2*-Cre causes fertility defects and the development of ovarian granulosa cell tumors, with no uterine phenotype reported [68]. Interestingly, triple deletion of *Smad1*, *Smad5*, and *Smad4* using the same Cre leads to defects in oviductal and myometrial development and blastocyst implantation [69]. Furthermore, expression of genes associated with oviductal development and cell differentiation is impaired in *Smad1/5/4 Amhr2*-Cre cKO mice [69]. *Smad1/5/4 Amhr2*-Cre cKO mice also show partially compromised decidualization, which may be caused by dysregulation of decidualization-associated genes such as *Bmp2*, *Wnt4*, and *Ptgs2* [69]. These studies suggest a complex role of SMAD signaling in uterine decidualization.

### Evidence from human studies

Supporting a role for TGFβ signaling in uterine decidualization in humans, the expression of a number of TGFβ family ligands including BMP2, BMP4, BMP7, GDF5, GDF8, and GDF11 is detectable in the secretory phase human endometrium and cultured human ESCs [70]. Decidual cells also express BMP2, GDF5, and TGFβ1 [70]. Although it has been long studied, the functional role of TGFβ1 in human decidualization remains controversial. It has been shown that TGFβ1 reduces the expression of PRL, IGFBP-1, and tissue factor (TF) in human ESCs, suggesting an inhibitory role of TGFβ1 in decidualization [71]. Further studies revealed the involvement of SMAD-dependent and SMAD-independent pathways in TGFβ1 inhibition of PRL and IGFBP-1 expression, respectively [71]. Moreover, TGFβ1 inhibits the expression of PGR and WNT antagonist Dickkopf-1 (DKK) in differentiated ESCs via the respective SMAD-dependent and SMAD-independent mechanisms [72]. In contrast to the inhibitory role of TGFβ1 in decidualization, other investigators have demonstrated that the secretion of TGFβ1 increases during in vitro decidualization of human ESCs, and that recombinant TGFβ1 promotes the decidualization process [70, 73, 74]. The reason for the contradictory effects of TGFβ1 on ESC decidualization is not known, but may be associated with differences in experimental conditions utilized in different studies.

Strong evidence supports the implication of BMP signaling in human ESC decidualization. The aforementioned role of BMP2 in uterine decidualization in mice has been reinforced by studies using in vitro cultured human ESCs, where secretion of BMP2 is increased during decidualization and recombinant BMP2 protein stimulates the decidual response [70]. A similar BMP2-WNT4 signaling mechanism may operate during decidualization in human and mouse ESCs [53]; downregulation of *WNT4* hampers BMP2-induced differentiation while overexpression of *WNT4* promotes cell differentiation [75]. The receptors that mediate BMP2 signaling during ESC decidualization have not been well defined. However, knockdown of expression of the BMP type 1 receptor, ACVR1, in human ESCs impairs the expression of decidualization markers [59]. Further experiments using chromatin immunoprecipitation revealed the importance of the BMP-ACVR1-SMAD1/5-CEBPB-PGR signaling axis in human ESC decidualization [59]. Unlike BMP2, BMP7 reduces E2/P4-induced expression of IGFBP1 mRNA in human ESCs in culture, suggesting an inhibitory role of BMP7 in ESC decidualization [76]. This finding seems to contradict results from the mouse model in which loss of BMP7 affects decidual gene expression [52]. Expression analysis has shown that the levels of BMPR1A, BMPR1B and BMPR2 are lower in leiomyoma-associated endometrium that secretes high levels of TGFβ3 versus normal endometrium [77]. Furthermore, treatment of ESCs with TGFβ3 reduces the expression of BMP receptors, suggesting that TGFβ signaling may be intertwined with BMP signaling in regulating functions of uterine decidual cells [77]. Further experiments are needed to determine potential interactions between TGFβs and BMPs during uterine decidualization.

There is also considerable evidence for the involvement of activins in human decidualization [78]. Activin receptors in the stromal and endothelial cells of human endometria are highly expressed during the early secretory phase of the menstrual cycle and early pregnancy [79]. Furthermore, activin A dose-dependently increases the production of PRL, and the effect can be attenuated by follistatin in decidualizing ESCs [80]. In combination with the fact that decidual cells secrete dimeric activin A, these findings point to an autocrine/paracrine action of activin A in human decidualization [80]. Furthermore, a potential link between activin A and the production of matrix metalloproteinases and IL11 in the mechanism of decidualization has been suggested [81, 82]. In addition, concentrations of activin B in serum are lower in ectopic pregnancies containing less decidualized stroma versus intrauterine pregnancies; and decidualization of uterine stromal cells is accompanied by upregulation of expression of inhibin/activin beta-B [83]. Thus, both activins A and B are plausible regulators of decidualization in human ESCs.

Consistent with the inhibitory role of LEFTY in uterine decidualization in mice, overexpression of LEFTY1 in human ESCs impairs their secretion of PRL and IGFBP1 [84]. Studies using human uterine fibroblast cells also support an inhibitory function of LEFTY in uterine decidualization, with the involvement of key transcription factors, FOXO1 and ETS proto-oncogene 1 (ETS1) [58]. These findings led to the suggestion that LEFTY may serve as a molecular switch controlling stromal cell differentiation and decidual reprogramming during early pregnancy [58].

## Conclusion and future directions

The application of Cre-LoxP technology has accelerated the generation of new knowledge and understanding of the functions of TGFβ signaling in decidualization, a key event associated with implantation and development of blastocysts/conceptuses (Fig. 1b). Despite that advancement in knowledge, the functional signaling circuitries among ligands, receptors, and SMADs remain to be elucidated. Future studies are warranted to not only define the signaling landscape, but also unravel the functional interactions among TGFβ signaling circuitries. For instance, it has been reported that PI3K/AKT, ERK, and JNK are regulators of decidualization [85–88] and some studies have suggested that PI3K/AKT signaling activities are downregulated during decidualization [87, 88]. Little is known about the role of TGFβ-activated kinase 1 (TAK1) in uterine decidualization. It is also not clear whether those non-canonical TGFβ signaling elements are also activated by TGFβ superfamily proteins in the context of uterine decidualization (Fig. 1b). If so, how are their functions orchestrated to fulfill the program of differentiation of endometrial cells? In addition, it remains challenging to delineate the functional ligands-receptor-SMAD/non-SMAD pathways and signaling crosstalk on the roadmap to decidualization.

Non-coding RNAs and epigenetic modifications are emerging regulators of uterine decidualization. MicroRNAs (miRNAs), non-coding RNAs that are ~22 nt long transcript, play important roles in post-transcriptional gene regulation [89]. Recent findings point to a likely role for non-coding RNAs in blastocyst implantation, uterine development, decidualization, and myometrial function [90–93]. For example, the levels of miR-542-3p are lower in decidualizing versus normal human ESCs, and overexpression of miR-542-3p inhibits the expression of *IGFBP1*, *PRL,* and *WNT4*, suggesting an inhibitory role of miR-542-3p in decidualization [94]. It has also been reported that miR-181b-5p regulates the expression of cell migration associated proteins during decidualization [95]. Although TGFβ signaling regulates miRNA biosynthesis/expression [96–98], little is known about interactions between TGFβ signaling and miRNAs in the regulation of decidualization. Future efforts are needed to gain a comprehensive understanding of the role of TGFβ-associated non-coding RNAs in uterine decidualization.

DNA/histone methylation appears to be involved in uterine decidualization. DNA methylation at cytosines represents a common epigenetic modification of genes. Our understanding of DNA methylation in decidualization is just beginning [99]. Recent studies have shown that DNA methyltransferase 1 (*Dnmt1*) and *Dnmt3a* are expressed in mouse ESCs during early pregnancy [100]. Treatment of mice with the DNA methyltransferase inhibitor 5-aza-2-deoxycytodine (5-aza-dC) during the peri- or postimplantation period impairs uterine decidualization [100]. Histone methylation, an important post-translational modification, adds methyl groups to specific amino acids of histones. Enhancer of zeste homolog 2 (EZH2), a histone methyltransferase, represses gene transcription by tri-methylation of lysine 27 on H3 histones (H3K27me3) [101]. The expression of EZH2 mRNA and protein is reduced in cultured human decidualizing cells induced by 8-bromo-cAMP and/or medroxyprogesterone acetate (MPA), as is associated with loss of H3K27me3 in the proximal promoters of *PRL* and *IGFBP1* [102]. Meanwhile, a transcriptionally permissive chromatin seems to be established due to the loss of H3K27me3 and enrichment in acetylation of H3K27 [102]. The outcome of such a chromatic remodeling is the phenotypic switch of ESCs from proliferation to decidualization [102]. As further evidence, chromobox 4 (CBX4)/ring finger protein 2 (RNF2/Ring1B) containing polycomb repressive complex 1 (PRC1) is an important regulator of decidualization in mice [103]. Of note, TGFβ superfamily members regulate EZH2 expression [104]. Therefore, it is imperative to determine whether TGFβ signaling and epigenetic programming are linked to event responsible for uterine decidualization during pregnancy.

In summary, further understanding TGFβ superfamily signaling associated cellular, molecular, and epigenetic mechanisms underlying decidualization is needed. In particular, deciphering the interrelationship among TGFβ signaling circuitries and their potential interactions with epigenetic modifications/non-coding RNAs may prove useful in developing novel therapeutic strategies for the treatment of uterine disorders associated with deficiencies in decidualization.

## References

[1] Matzuk MM, Li Q, Coticchio G, Albertini DF, De Santis L (2013). How the oocyte influences follicular cell function and why. Oogenesis.

[2] Fang X, Gao Y, Li Q (2016). SMAD3 activation: a converging point of dysregulated TGF-Beta superfamily signaling and genetic aberrations in granulosa cell tumor development?. Biol Reprod.

[3] Derynck R, Zhang YE (2003). Smad-dependent and Smad-independent pathways in TGF-beta family signalling. Nature.

[4] Wang ZP, Mu XY, Guo M, Wang YJ, Teng Z, Mao GP, Niu WB, Feng LZ, Zhao LH, Xia GL (2014). Transforming growth factor-beta signaling participates in the maintenance of the primordial follicle pool in the mouse ovary. J Biol Chem.

[5] Hussein TS, Thompson JG, Gilchrist RB (2006). Oocyte-secreted factors enhance oocyte developmental competence. Dev Biol.

[6] Gilchrist RB, Lane M, Thompson JG (2008). Oocyte-secreted factors: regulators of cumulus cell function and oocyte quality. Hum Reprod Update.

[7] Pangas SA, Li X, Robertson EJ, Matzuk MM (2006). Premature luteinization and cumulus cell defects in ovarian-specific Smad4 knockout mice. Mol Endocrinol.

[8] Tomic D, Miller KP, Kenny HA, Woodruff TK, Hoyer P, Flaws JA (2004). Ovarian follicle development requires Smad3. Mol Endocrinol.

[9] Li Q, Pangas SA, Jorgez CJ, Graff JM, Weinstein M, Matzuk MM (2008). Redundant roles of SMAD2 and SMAD3 in ovarian granulosa cells in vivo. Mol Cell Biol.

[10] Yu C, Zhang YL, Fan HY (2013). Selective Smad4 knockout in ovarian preovulatory follicles results in multiple defects in ovulation. Mol Endocrinol.

[11] Pangas SA, Jorgez CJ, Tran M, Agno J, Li X, Brown CW, Kumar TR, Matzuk MM (2007). Intraovarian activins are required for female fertility. Mol Endocrinol.

[12] Richards JS, Pangas SA (2010). The ovary: basic biology and clinical implications. J Clin Invest.

[13] Pangas SA (2012). Regulation of the ovarian reserve by members of the transforming growth factor beta family. Mol Reprod Dev.

[14] Juengel JL, McNatty KP (2005). The role of proteins of the transforming growth factor-beta superfamily in the intraovarian regulation of follicular development. Hum Reprod Update.

[15] Knight PG, Glister C (2006). TGF-beta superfamily members and ovarian follicle development. Reproduction.

[16] Trombly DJ, Woodruff TK, Mayo KE (2009). Roles for transforming growth factor beta superfamily proteins in early folliculogenesis. Semin Reprod Med.

[17] Bazer FW, Wu G, Spencer TE, Johnson GA, Burghardt RC, Bayless K (2010). Novel pathways for implantation and establishment and maintenance of pregnancy in mammals. Mol Hum Reprod.

[18] Burghardt RC, Johnson GA, Jaeger LA, Ka H, Garlow JE, Spencer TE, Bazer FW (2002). Integrins and extracellular matrix proteins at the maternal-fetal interface in domestic animals. Cells Tissues Organs.

[19] Garcia EV, Hamdi M, Barrera AD, Sanchez-Calabuig MJ, Gutierrez-Adan A, Rizos D (2017). Bovine embryo-oviduct interaction in vitro reveals an early cross talk mediated by BMP signaling. Reproduction.

[20] Li G, Khateeb K, Schaeffer E, Zhang B, Khatib H (2012). Genes of the transforming growth factor-beta signalling pathway are associated with pre-implantation embryonic development in cattle. J Dairy Res.

[21] Rappolee DA, Brenner CA, Schultz R, Mark D, Werb Z (1988). Developmental expression of PDGF, TGF-alpha, and TGF-beta genes in preimplantation mouse embryos. Science.

[22] de Mochel NSR, Luong M, Chiang M, Javier AL, Luu E, Fujimori T, MacGregor GR, Cinquin O, Cho KWY (2015). BMP signaling is required for cell cleavage in preimplantation-mouse embryos. Dev Biol.

[23] Wu MY, Hill CS (2009). Tgf-beta superfamily signaling in embryonic development and homeostasis. Dev Cell.

[24] Kitisin K, Saha T, Blake T, Golestaneh N, Deng M, Kim C, Tang Y, Shetty K, Mishra B, Mishra L (2007). Tgf-Beta signaling in development. Sci STKE.

[25] Mummery CL (2001). Transforming growth factor beta and mouse development. Microsc Res Tech.

[26] Mullen RD, Behringer RR (2014). Molecular genetics of Mullerian duct formation, regression and differentiation. Sex Dev.

[27] Stewart CA, Behringer RR (2012). Mouse oviduct development. Results Probl Cell Differ.

[28] Monsivais D, Matzuk MM, Pangas SA. The TGF-beta family in the reproductive tract**.** Cold Spring Harb Perspect Biol*.* 2017**:**pii: a022251. 10.1101/cshperspect.a022251 PMC5554737 28193725

[29] Li Q, Agno JE, Edson MA, Nagaraja AK, Nagashima T, Matzuk MM (2011). Transforming growth factor beta receptor type 1 is essential for female reproductive tract integrity and function. PLoS Genet.

[30] Gao Y, Bayless KJ, Li Q (2014). TGFBR1 is required for mouse myometrial development. Mol Endocrinol.

[31] Gao Y, Li S, Li Q (2014). Uterine epithelial cell proliferation and endometrial hyperplasia: evidence from a mouse model. Mol Hum Reprod.

[32] Young JC, Wakitani S, Loveland KL (2015). TGF-beta superfamily signaling in testis formation and early male germline development. Semin Cell Dev Biol.

[33] Maruyama T, Yoshimura Y (2008). Molecular and cellular mechanisms for differentiation and regeneration of the uterine endometrium. Endocr J.

[34] Ramathal CY, Bagchi IC, Taylor RN, Bagchi MK (2010). Endometrial decidualization: of mice and men. Semin Reprod Med.

[35] Abrahamsohn PA, Zorn TM (1993). Implantation and decidualization in rodents. J Exp Zool.

[36] Telgmann R, Gellersen B (1998). Marker genes of decidualization: activation of the decidual prolactin gene. Hum Reprod Update.

[37] Croze F, Kennedy TG, Schroedter IC, Friesen HG, Murphy LJ (1990). Expression of insulin-like growth factor-I and insulin-like growth factor-binding protein-1 in the rat uterus during decidualization. Endocrinology.

[38] Large MJ, DeMayo FJ (2012). The regulation of embryo implantation and endometrial decidualization by progesterone receptor signaling. Mol Cell Endocrinol.

[39] Wetendorf M, DeMayo FJ (2012). The progesterone receptor regulates implantation, decidualization, and glandular development via a complex paracrine signaling network. Mol Cell Endocrinol.

[40] Lee K, Jeong J, Kwak I, Yu CT, Lanske B, Soegiarto DW, Toftgard R, Tsai MJ, Tsai S, Lydon JP, DeMayo FJ (2006). Indian hedgehog is a major mediator of progesterone signaling in the mouse uterus. Nat Genet.

[41] Lee KY, Jeong JW, Wang J, Ma L, Martin JF, Tsai SY, Lydon JP, DeMayo FJ (2007). Bmp2 is critical for the murine uterine decidual response. Mol Cell Biol.

[42] Kurihara I, Lee DK, Petit FG, Jeong J, Lee K, Lydon JP, DeMayo FJ, Tsai MJ, Tsai SY (2007). COUP-TFII mediates progesterone regulation of uterine implantation by controlling ER activity. PLoS Genet.

[43] Franco HL, Dai D, Lee KY, Rubel CA, Roop D, Boerboom D, Jeong JW, Lydon JP, Bagchi IC, Bagchi MK, DeMayo FJ (2011). WNT4 is a key regulator of normal postnatal uterine development and progesterone signaling during embryo implantation and decidualization in the mouse. FASEB J.

[44] Li Q, Kannan A, DeMayo FJ, Lydon JP, Cooke PS, Yamagishi H, Srivastava D, Bagchi MK, Bagchi IC (2011). The antiproliferative action of progesterone in uterine epithelium is mediated by Hand2. Science.

[45] Vasquez YM, Mazur EC, Li XL, Kommagani R, Jiang LC, Chen R, Lanz RB, Kovanci E, Gibbons WE, DeMayo FJ (2015). FOXO1 is required for binding of PR on IRF4, novel transcriptional regulator of endometrial stromal decidualization. Mol Endocrinol.

[46] Wang W, Taylor RN, Bagchi IC, Bagchi MK (2012). Regulation of human endometrial stromal proliferation and differentiation by C/EBP beta involves cyclin E-cdk2 and STAT3. Mol Endocrinol.

[47] Godbole G, Modi D (2010). Regulation of decidualization, interleukin-11 and interleukin-15 by homeobox a 10 in endometrial stromal cells. J Reprod Immunol.

[48] Lim H, Ma L, Ma WG, Maas RL, Dey SK (1999). Hoxa-10 regulates uterine stromal cell responsiveness to progesterone during implantation and decidualization in the mouse. Mol Endocrinol.

[49] Hofmann AP, Gerber SA, Croy BA (2014). Uterine natural killer cells pace early development of mouse decidua basalis. Mol Hum Reprod.

[50] Shooner C, Caron PL, Frechette-Frigon G, Leblanc V, Dery MC, Asselin E (2005). TGF-beta expression during rat pregnancy and activity on decidual cell survival. Reprod Biol Endocrinol.

[51] Caron PL, Frechette-Frigon G, Shooner C, Leblanc V, Asselin E (2009). Transforming growth factor beta isoforms regulation of Akt activity and XIAP levels in rat endometrium during estrous cycle, in a model of pseudopregnancy and in cultured decidual cells. Reprod Biol Endocrinol.

[52] Monsivais D, Clementi C, Peng J, Fullerton PT, Prunskaite-Hyyrylainen R, Vainio SJ, Matzuk MM (2017). BMP7 induces uterine receptivity and blastocyst attachment. Endocrinology.

[53] Li Q, Kannan A, Wang W, Demayo FJ, Taylor RN, Bagchi MK, Bagchi IC (2007). Bone morphogenetic protein 2 functions via a conserved signaling pathway involving Wnt4 to regulate uterine decidualization in the mouse and the human. J Biol Chem.

[54] Fullerton PT, Monsivais D, Kommagani R, Matzuk MM (2017). Follistatin is critical for mouse uterine receptivity and decidualization. Proc Natl Acad Sci U S A.

[55] Park CB, Dufort D (2013). NODAL signaling components regulate essential events in the establishment of pregnancy. Reproduction.

[56] Park CB, DeMayo FJ, Lydon JP, Dufort D (2012). NODAL in the uterus is necessary for proper placental development and maintenance of pregnancy. Biol Reprod.

[57] Tang M, Mikhailik A, Pauli I, Giudice LC, Fazelabas AT, Tulac S, Carson DD, Kaufman DG, Barbier C, Creemers JW, Tabibzadeh S (2005). Decidual differentiation of stromal cells promotes Proprotein Convertase 5/6 expression and lefty processing. Endocrinology.

[58] Tang M, Naidu D, Hearing P, Handwerger S, Tabibzadeh S (2010). LEFTY, a member of the transforming growth factor-beta superfamily, inhibits uterine stromal cell differentiation: a novel autocrine role. Endocrinology.

[59] Clementi C, Tripurani SK, Large MJ, Edson MA, Creighton CJ, Hawkins SM, Kovanci E, Kaartinen V, Lydon JP, Pangas SA (2013). Activin-like kinase 2 functions in peri-implantation uterine signaling in mice and humans. PLoS Genet.

[60] Monsivais D, Clementi C, Peng J, Titus MM, Barrish JP, Creighton CJ, Lydon JP, DeMayo FJ, Matzuk MM (2016). Uterine ALK3 is essential during the window of implantation. Proc Natl Acad Sci U S A.

[61] Edson MA, Nalam RL, Clementi C, Franco HL, Demayo FJ, Lyons KM, Pangas SA, Matzuk MM (2010). Granulosa cell-expressed BMPR1A and BMPR1B have unique functions in regulating fertility but act redundantly to suppress ovarian tumor development. Mol Endocrinol.

[62] Yi SE, LaPolt PS, Yoon BS, Chen JYC, Lu JKH, Lyons KM (2001). The type I BMP receptor BmprIB is essential for female reproductive function. Proc Natl Acad Sci U S A.

[63] Nagashima T, Li Q, Clementi C, Lydon JP, Demayo FJ, Matzuk MM (2013). BMPR2 is required for postimplantation uterine function and pregnancy maintenance. J Clin Invest.

[64] Peng J, Monsivais D, You R, Zhong H, Pangas SA, Matzuk MM (2015). Uterine activin receptor-like kinase 5 is crucial for blastocyst implantation and placental development. Proc Natl Acad Sci U S A.

[65] Reissmann E, Jornvall H, Blokzijl A, Andersson O, Chang C, Minchiotti G, Persico MG, Ibanez CF, Brivanlou AH (2001). The orphan receptor ALK7 and the Activin receptor ALK4 mediate signaling by nodal proteins during vertebrate development. Genes Dev.

[66] Peng J, Fullerton PT, Monsivais D, Clementi C, Su GH, Matzuk MM (2015). Uterine activin-like kinase 4 regulates trophoblast development during mouse placentation. Mol Endocrinol.

[67] Zhao KQ, Lin HY, Zhu C, Yang X, Wang H (2012). Maternal Smad3 deficiency compromises decidualization in mice. J Cell Biochem.

[68] Pangas SA, Li X, Umans L, Zwijsen A, Huylebroeck D, Gutierrez C, Wang D, Martin JF, Jamin SP, Behringer RR (2008). Conditional deletion of Smad1 and Smad5 in somatic cells of male and female gonads leads to metastatic tumor development in mice. Mol Cell Biol.

[69] Rodriguez A, Tripurani SK, Burton JC, Clementi C, Larina I, Pangas SA (2016). SMAD signaling is required for structural integrity of the female reproductive tract and uterine function during early pregnancy in mice. Biol Reprod.

[70] Stoikos CJ, Harrison CA, Salamonsen LA, Dimitriadis E (2008). A distinct cohort of the TGFbeta superfamily members expressed in human endometrium regulate decidualization. Hum Reprod.

[71] Kane NM, Jones M, Brosens JJ, Kelly RW, Saunders PT, Critchley HO (2010). TGFbeta1 attenuates expression of prolactin and IGFBP-1 in decidualized endometrial stromal cells by both SMAD-dependent and SMAD-independent pathways. PLoS One.

[72] Kane N, Jones M, Brosens JJ, Saunders PTK, Kelly RW, Critchley HOD (2008). Transforming growth factor-beta 1 attenuates expression of both the progesterone receptor and Dickkopf in differentiated human endometrial stromal cells. Mol Endocrinol.

[73] Chang HJ, Lee JH, Hwang KJ, Kim MR, Chang KH, Park DW, Min CK (2008). Transforming growth factor (TGF)-beta 1-induced human endometrial stromal cell decidualization through extracellular signal-regulated kinase and Smad activation in vitro: peroxisome proliferator-activated receptor gamma acts as a negative regulator of TGF-beta 1. Fertil Steril.

[74] Kim MR, Park DW, Lee JH, Choi DS, Hwang KJ, Ryu HS, Min CK (2005). Progesterone-dependent release of transforming growth factor-beta1 from epithelial cells enhances the endometrial decidualization by turning on the Smad signalling in stromal cells. Mol Hum Reprod.

[75] Li Q, Kannan A, Das A, Demayo FJ, Hornsby PJ, Young SL, Taylor RN, Bagchi MK, Bagchi IC (2013). WNT4 acts downstream of BMP2 and functions via beta-catenin signaling pathway to regulate human endometrial stromal cell differentiation. Endocrinology.

[76] Kodama A, Yoshino O, Osuga Y, Harada M, Hasegawa A, Hamasaki K, Takamura M, Koga K, Hirota Y, Hirata T (2010). Progesterone decreases bone morphogenetic protein (BMP) 7 expression and BMP7 inhibits decidualization and proliferation in endometrial stromal cells. Hum Reprod.

[77] Sinclair DC, Mastroyannis A, Taylor HS (2011). Leiomyoma simultaneously impair endometrial BMP-2-mediated decidualization and anticoagulant expression through secretion of TGF-beta 3. J Clin Endocrinol Metab.

[78] Salamonsen LA, Dimitriadis E, Jones RL, Nie G (2003). Complex regulation of decidualization: a role for cytokines and proteases--a review. Placenta.

[79] Jones RL, Salamonsen LA, Zhao YC, Ethier JF, Drummond AE, Findlay JK (2002). Expression of activin receptors, follistatin and betaglycan by human endometrial stromal cells; consistent with a role for activins during decidualization. Mol Hum Reprod.

[80] Jones RL, Salamonsen LA, Findlay JK (2002). Activin a promotes human endometrial stromal cell decidualization in vitro. J Clin Endocrinol Metab.

[81] Jones RL, Findlay JK, Farnworth PG, Robertson DM, Wallace E, Salamonsen LA (2006). Activin a and inhibin a differentially regulate human uterine matrix metalloproteinases: potential interactions during decidualization and trophoblast invasion. Endocrinology.

[82] Menkhorst E, Salamonsen LA, Zhang J, Harrison CA, Gu J, Dimitriadis E (2010). Interleukin 11 and activin a synergise to regulate progesterone-induced but not cAMP-induced decidualization. J Reprod Immunol.

[83] Horne AW, van den Driesche S, King AE, Burgess S, Myers M, Ludlow H, Lourenco P, Ghazal P, Williams AR, Critchley HOD, Duncan WC (2008). Endometrial inhibin/activin beta-B subunit expression is related to decidualization and is reduced in tubal ectopic pregnancy. J Clin Endocrinol Metab.

[84] Li H, Li H, Bai L, Yu H (2014). Lefty inhibits in vitro decidualization by regulating P57 and cyclin D1 expressions. Cell Biochem Funct.

[85] Toyofuku A, Hara T, Taguchi T, Katsura Y, Ohama K, Kudo Y (2006). Cyclic and characteristic expression of phosphorylated Akt in human endometrium and decidual cells in vivo and in vitro. Hum Reprod.

[86] Zhou WJ, Hou XX, Wang XQ, Li DJ. Fibroblast growth factor 7 regulates proliferation and decidualization of human endometrial stromal cells via ERK and JNK pathway in an autocrine manner. Reprod Sci. 2017. doi:10.1177/1933719117697122. 10.1177/1933719117697122 28270036

[87] Yin X, Pavone ME, Lu Z, Wei J, Kim JJ (2012). Increased activation of the PI3K/AKT pathway compromises decidualization of stromal cells from endometriosis. J Clin Endocrinol Metab.

[88] Fabi F, Grenier K, Parent S, Adam P, Tardif L, Leblanc V, Asselin E (2017). Regulation of the PI3K/Akt pathway during decidualization of endometrial stromal cells. PLoS One.

[89] Bartel DP (2004). MicroRNAs: genomics, biogenesis, mechanism, and function. Cell.

[90] Galliano D, Pellicer A (2014). MicroRNA and implantation. Fertil Steril.

[91] Dior UP, Kogan L, Chill HH, Eizenberg N, Simon A, Revel A (2014). Emerging roles of microRNA in the embryo-endometrium cross talk. Semin Reprod Med.

[92] Nothnick WB (2016). Non-coding RNAs in uterine development, function and disease. Adv Exp Med Biol.

[93] Renthal NE, Williams KC, Mendelson CR (2013). MicroRNAs-mediators of myometrial contractility during pregnancy and labour. Nat Rev Endocrinol.

[94] Tochigi H, Kajihara T, Mizuno Y, Mizuno Y, Tamaru S, Kamei Y, Okazaki Y, Brosens JJ, Ishihara O (2017). Loss of miR-542-3p enhances IGFBP-1 expression in decidualizing human endometrial stromal cells. Sci Rep.

[95] Graham A, Holbert J, Nothnick WB (2017). miR-181b-5p modulates cell migratory proteins, tissue inhibitor of metalloproteinase 3, and annexin A2 during in vitro decidualization in a human endometrial stromal cell line. Reprod Sci.

[96] Davis BN, Hilyard AC, Lagna G, Hata A (2008). SMAD proteins control DROSHA-mediated microRNA maturation. Nature.

[97] Blahna MT, Hata A (2012). Smad-mediated regulation of microRNA biosynthesis. FEBS Lett.

[98] Liu Y, Li Y, Li N, Teng W, Wang M, Zhang Y, Xiao Z (2016). TGF-beta1 promotes scar fibroblasts proliferation and transdifferentiation via up-regulating MicroRNA-21. Sci Rep.

[99] Gao F, Das SK (2014). Epigenetic regulations through DNA methylation and hydroxymethylation: clues for early pregnancy in decidualization. Biomol Concepts.

[100] Gao F, Ma XH, Rusie A, Hemingway J, Ostmann AB, Chung D, Das SK (2012). Epigenetic changes through DNA methylation contribute to uterine stromal cell decidualization. Endocrinology.

[101] Cao R, Wang L, Wang H, Xia L, Erdjument-Bromage H, Tempst P, Jones RS, Zhang Y (2002). Role of histone H3 lysine 27 methylation in Polycomb-group silencing. Science.

[102] Grimaldi G, Christian M, Steel JH, Henriet P, Poutanen M, Brosens JJ (2011). Down-regulation of the histone methyltransferase EZH2 contributes to the epigenetic programming of decidualizing human endometrial stromal cells. Mol Endocrinol.

[103] Bian F, Gao F, Kartashov AV, Jegga AG, Barski A, Das SK (2016). Polycomb repressive complex 1 controls uterine decidualization. Sci Rep.

[104] Wang L, Xu X, Cao Y, Li Z, Cheng H, Zhu G, Duan F, Na J, Han JJ, Chen YG (2017). Activin/Smad2-induced histone H3 lys-27 trimethylation (H3K27me3) reduction is crucial to initiate mesendoderm differentiation of human embryonic stem cells. J Biol Chem.

[105] Myers M, Middlebrook BS, Matzuk MM, Pangas SA (2009). Loss of inhibin alpha uncouples oocyte-granulosa cell dynamics and disrupts postnatal folliculogenesis. Dev Biol.

[106] Feinberg RF, Kliman HJ, Wang CL (1994). Transforming growth factor-beta stimulates trophoblast oncofetal fibronectin synthesis in vitro: implications for trophoblast implantation in vivo. J Clin Endocrinol Metab.

[107] Chang H, Brown CW, Matzuk MM (2002). Genetic analysis of the mammalian transforming growth factor-β superfamily. Endocr Rev.

[108] Kleiter I, Song J, Lukas D, Hasan M, Neumann B, Croxford AL, Pedre X, Hovelmeyer N, Yogev N, Mildner A (2010). Smad7 in T cells drives T helper 1 responses in multiple sclerosis and experimental autoimmune encephalomyelitis. Brain.

[109] Petit FG, Deng C, Jamin SP (2016). Partial mullerian duct retention in Smad4 conditional mutant male mice. Int J Biol Sci.

[110] Orvis GD, Jamin SP, Kwan KM, Mishina Y, Kaartinen VM, Huang S, Roberts AB, Umans L, Huylebroeck D, Zwijsen A (2008). Functional redundancy of TGF-beta family type I receptors and receptor-Smads in mediating anti-Mullerian hormone-induced Mullerian duct regression in the mouse. Biol Reprod.

